# Potential Synergy Activity of the Novel Ceragenin, CSA-13, against Carbapenem-Resistant *Acinetobacter baumannii* Strains Isolated from Bacteremia Patients

**DOI:** 10.1155/2014/710273

**Published:** 2014-03-24

**Authors:** Cagla Bozkurt-Guzel, Paul B. Savage, Alper Akcali, Berna Ozbek-Celik

**Affiliations:** ^1^Department of Pharmaceutical Microbiology, Faculty of Pharmacy, Istanbul University, Beyazit, 34116 Istanbul, Turkey; ^2^Department of Chemistry and Biochemistry, Brigham Young University, Provo, UT 84602, USA; ^3^Department of Medical Microbiology, Faculty of Medicine, Canakkale Onsekiz Mart University, 17100 Canakkale, Turkey

## Abstract

Carbapenem-resistant *Acinetobacter baumannii* is an important cause of nosocomial infections, particularly in patients in the intensive care units. As chronic infections are difficult to treat, attempts have been made to discover new antimicrobials. Ceragenins, designed to mimic the activities of antimicrobial peptides, are a new class of antimicrobial agents. In this study, the in vitro activities of CSA-13 either alone or in combination with colistin (sulphate), tobramycin, and ciprofloxacin were investigated using 60 carbapenem-resistant *A. baumannii* strains isolated from bacteremia patients blood specimens. MICs and MBCs were determined by microbroth dilution technique. Combinations were assessed by using checkerboard technique. The MIC_50_ values (mg/L) of CSA-13, colistin, tobramycin, and ciprofloxacin were 2, 1, 1.25, and 80, respectively. The MIC_90_ (mg/L) of CSA-13 and colistin were 8 and 4. The MBCs were equal to or twice greater than those of the MICs. Synergistic interactions were mostly seen with CSA-13-colistin (55%), whereas the least synergistic interactions were observed in the CSA-13-tobramycin (35%) combination. No antagonism was observed. CSA-13 appears to be a good candidate for further investigations in the treatment of *A. baumannii* infections. However, future studies should be performed to correlate the safety, efficacy, and pharmacokinetic parameters of this molecule.

## 1. Introduction

*Acinetobacter baumannii* is a Gram-negative coccobacillus that recently has become one of the most common and highly antibiotic resistant pathogens throughout the world, and it is associated with high rates of morbidity and mortality [1, 2]. The most common clinical manifestations of* A. baumannii* infections in the intensive care units (ICUs) are ventilator associated pneumonia (VAP) and bacteremia, which are associated with morbidity and mortality rates as high as 52% [3, 4]. Invariably, one of the most alarming characteristics of this microorganism is its ability to manifest resistance to all available antibiotics including carbapenems, which is even higher than in other Gram-negative bacilli included in the ESKAPE group (*Enterococcus faecium, Staphylococcus aureus, Klebsiella pneumoniae, A. baumannii, Pseudomonas aeruginosa,* and* Enterobacter* species) [5]. Multidrug-resistant (MDR)* A. baumannii* is a growing threat that leaves few therapeutic options and recently there has been a dramatic increase in carbapenem resistance in* A. baumannii*. The mechanisms of resistance to antimicrobials are principally acquired through its ability to exchange genetic material. This attribute makes the treatment of* A. baumannii* infections particularly difficult, especially in certain types of infections [6]. The lack of new antibiotics to treat MDR* A. baumannii* infections has led the Infectious Disease Society of America (IDSA) to describe* A. baumannii* as “an emblematic case of the mismatch between unmet medical needs and the current antimicrobial research and development pipeline” [7].

As chronic infections are difficult to treat, attempts have been made to discover new antimicrobial agents targeting novel sites that may circumvent resistance. One frequently studied target is the bacterial membrane. Most antimicrobial peptides display broad-spectrum antibacterial activities and target the bacterial membrane. However, many antimicrobial peptides are difficult to synthesize and purify due to their complexity and size [8]. In addition, antimicrobial peptides can be substrates for proteases, which limit their in vivo half-lives [9]. Consequently, development of nonpeptide mimics of antimicrobial peptides may provide a means of using the antimicrobial strategies evolved over eons without the disadvantages of peptide therapeutics.

Recently, a series of cationic derivatives of cholic acid have been synthesized and have been found to have properties that may make them useful antimicrobial agents. The ceragenins, designed to mimic the activities of antimicrobial peptides, are a new class of antimicrobial agent. Ceragenins are not peptide based, are not salt sensitive, and are relatively simple to prepare and purify on a large scale [10]. Among them, CSA-13, which stands for cationic steroidal antimicrobial, is a lead ceragenin and is highly active against Gram-positive and Gram-negative bacteria. MIC determinations against common Gram-positive and Gram-negative bacteria have demonstrated that CSA-13 displays a broad spectrum of activity. CSA-13 displays antimicrobial activity against vancomycin-resistant* Staphylococcus aureus *[11],* Pseudomonas aeruginosa* [12, 13],* Helicobacter pylori* [14], and periodontopathic bacteria such as* Streptococcus mutans* and* Porphyromonas species* [15]. CSA-13 is also active against vaccinia virus [16] and* Trypanosoma cruzi* [17]. In animal studies, CSA-13 shows low toxicity, supporting this compound's possible application in human treatment [18].

In the setting of increasing resistance and diminishing therapeutic options, the “old” antibiotic colistin (polymyxin E) is now being used more extensively, especially in* P. aeruginosa* and carbapenem-resistant* A. baumannii* infections [19]. There are no current published studies evaluating the interactions between CSA-13 and colistin against carbapenem-resistant* A. baumannii* strains isolated from blood specimens. Therefore, the purpose of this study was to evaluate the in vitro activities of CSA-13 alone and in combination with colistin, tobramycin, and ciprofloxacin against 60 carbapenem-resistant* A. baumannii* strains isolated from bacteremia patients' blood specimens.

## 2. Materials and Methods

### 2.1. Bacterial Isolates

A total of sixty nonrepeat, bloodstream strains of carbapenem-resistant* A. baumannii* recovered from bacteremia patients in the year 2010-2011 admitted to the various hospitals in Turkey were included in the study. Thirty of these strains are obtained from the Department of Infectious Diseases and Clinical Microbiology, Medipol University, Istanbul, twenty of them are obtained from the Department of Infectious Diseases and Clinical Microbiology, Faculty of Medicine, Istanbul University, Istanbul, and the rest of them are obtained from Canakkale Onsekiz Mart University, Faculty of Medicine, Canakkale, Turkey. All strains were identified by the API 20 NE System (bioMerieux Vitek, Marcy l'Etoile, France). Isolates were defined as carbapenem-resistant strains using the disc diffusion and microdilution method. For the checkerboard experiments totally 20 strains from the three different institutions were used, since we carried out the combination experiments with susceptible strains.* Escherichia coli* ATCC 25922 (Rockville, Md., USA) was used as a quality control strain.

### 2.2. Antimicrobial Agents

**Figure 1:**
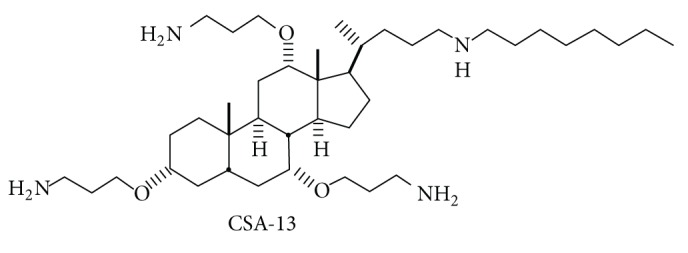
The chemical structure of ceragenin CSA-13 (molecular weight 822.94).

CSA-13 was synthesized from a cholic acid scaffold technique as previously described (Figure 1) [20]. Colistin was obtained from Sigma Aldrich and tobramycin, ciprofloxacin, and meropenem were kindly provided from Bilim and Bayer Pharmaceuticals and Astra Zeneca, respectively. Stock solutions from dry powders were prepared in water and stored frozen at −80°C. Frozen solutions of antibiotics were used within 6 months.

### 2.3. Media

Mueller-Hinton broth (MHB, Difco Laboratories, Detroit, MI) supplemented with divalent cations to a final concentration of 25 mg of Mg^2+^ and 50 mg of Ca^2+^ per liter (CSMHB) was used for all the experiments. Pour plates of Tryptic Soy agar (TSA, Difco Laboratories, Detroit, MI) were used for colony counts.

### 2.4. Determinations of MICs and MBCs

MICs were determined by the microbroth dilution technique as described by CLSI [21, 22]. Serial twofold dilutions ranging from 256 to 0.25 mg/L were prepared in CSMHB. The inoculum was prepared with a 4–6 h broth culture that gives a final concentration of 5 × 10^5^ cfu/mL in the test tray. Experiments were performed in duplicate. MBCs were determined at the conclusion of the incubation period by removing two 0.01 mL samples from each well demonstrating no visible growth and plated onto TSA. The MBC was defined as the lowest concentration of antibiotic giving at least a 99.9% killing of the initial inoculums [23].

### 2.5. Determination of Fractional Inhibitory Concentration Index (FICI)

The effects of antibiotics in combination were assessed by using the microbroth checkerboard technique [24]. Each microtiter well containing the mixture of antibiotics was inoculated with a 4–6 h broth culture diluted to give a final concentration of approximately 5 × 10^5^ cfu/mL. After incubation at 37°C for 18–20 h the fractional inhibitory concentration index (FICI) was determined as the combined concentration divided by the single concentration. The combination value was derived from the highest dilution of antibiotic combination permitting no visible growth. With this method, synergy was defined as a FICI of ≤0.5, no interaction as a FICI of >0.5–4, and antagonism as a FICI of 4.0 [25].

## 3. Results

### 3.1. Susceptibility

The in vitro activities of the studied antibiotics against 60* A. baumannii* strains are summarized in Table 1. Susceptibility testing demonstrated that the MIC ranges for CSA-13, colistin, tobramycin, and ciprofloxacin were 1–16, 0.06–32, 0.3–160, and 0.3–80 mg/L and MBC ranges for those antibiotics were 1–32, 0.06–32, 0.3–160, and 0.6–160 mg/L, respectively. As seen from the results, CSA-13 showed a similar pattern of MIC and MBC ranges as colistin. In addition, the highest MIC and MBC values of CSA-13 were just onefold higher of the MIC_90_ and MBC_90_ values. However, 14%, 55%, and 95% of the strains were found resistant to colistin, tobramycin, and ciprofloxacin, respectively. All the strains were resistant to meropenem. CSA-13 MICs (and also MBCs) of the colistin-resistant strains are at the same value or twofold greater than those of the colistin-resistant strains. There was no major difference between bactericidal and inhibitory endpoints. The MBCs were generally equal to or twofold greater than those of the MICs.

### 3.2. Checkerboard

The results of combination studies are shown in Table 2. With a FIC index of ≤0.5 as borderline, synergistic interactions were mostly seen with CSA-13-colistin combination (synergism was observed with 55% of the strains tested), whereas the least synergistic interactions were observed with the CSA-13-tobramycin combination (synergism was observed with 35% of the strains tested). No antagonism was observed with any combination.

## 4. Discussion

Ceragenins are a group of cholic acid derivatives that have potent activities against various microorganisms [10]. Here, we report an MIC_50_ of 2 mg/L for CSA-13 against 60 carbapenem-resistant* A. baumannii* strains. As seen from the results, MIC_90_ value of CSA-13 was equal to two dilutions higher of the MIC_50_ value, which is parallel to colistin results (Table 1). These results indicate that CSA-13 shows an activity with similar MIC values independent of whether or not the bacteria are resistant to other antibiotics. Probably, this situation could be attributed to its ability to permeabilize both outer and cytoplasmic membrane of the bacteria and its resistance to protease degradation [26]. These results support the idea that development of resistance to CSA-13 might be rare if it is used in the treatment. Our study also shows that CSA-13 has an MIC_50_/MBC_50_ ratio of 1, suggesting that the bactericidal activity is close to the inhibitory concentration. Indeed, varying CSA-13 concentrations at, below, and above the MIC demonstrated rapid bactericidal antimicrobial activity, even when the strains were resistant to colistin, ciprofloxacin, and/or tobramycin, similar to our previous work [13].

Carbapenem resistance rates are increasing to such an extent to threaten the world and this situation is becoming a routine phenotype for the* A. baumannii*. Therefore, in order to take the microorganism under control, selection of the antimicrobial agents is extremely important [27]. Increase of carbapenem resistance raises the fact that the reuse of old antibiotics like polymyxin E. (colistin) has a lower rate of mortality than carbapenems in treatment of multidrug resistance infections [19]. Colistin is frequently used to treat infections caused by carbapenem-resistant* A. baumannii*, due to its efficacy [28]. However, recently colistin resistance is reported worldwide, especially in Europe [29, 30]. In our country, Ergin et al. reported colistin resistance as 2% in* A. baumannii* [31]. According to our study, 86% of the strains were found to be colistin susceptible. Moreover, we demonstrated that of all the studied antibiotics, colistin was active at the lowest MIC (MIC_50_ = 1 *μ*g/mL) value against the strains. This may arise from the fact that colistin has been recently used for clinical use in Turkey.

Results of our study showed that the highest resistance rate is obtained with ciprofloxacin (95%). This was in accordance with one multicentric study [32]. High percentages of strains belonging to* A. baumannii* were resistant to ciprofloxacin, ofloxacin, and cefotaxime (79, 76, and 54%, resp.) by agar dilution method.

Management of the infections caused by carbapenem-resistant* A. baumannii* is difficult and combination therapy for the treatment of carbapenem-resistant* A. baumannii* has increasingly been used [28]. Therefore, in our study, in vitro interactions of CSA-13 in combination with colistin, tobramycin, and ciprofloxacin against carbapenem-resistant* A. baumannii* strains were assessed by using the microbroth checkerboard technique since it provides fast results and interpretation of these results is simple [24]. The results of this in vitro trial provide evidence that, with a FICI of ≤0.5 as borderline, synergistic interactions were detected in all combinations (Table 2). Synergistic interactions were mostly seen with CSA-13-colistin combination (55% of tested strains), whereas the least synergistic interactions were observed with the CSA-13-tobramycincombination (35% of tested strains).

Consequently, ceragenins are novel molecules resistant to proteolysis and promise opportunities in treatment of bacterial, fungal, and even viral infections. According to the results of this in vitro study, CSA-13 may have important therapeutic implications for infections caused by carbapenem-resistant* A. baumannii* strains. So, these molecules should be evaluated carefully and must be reserved for the most important necessity. Possible success for the combination therapy of ceragenins and colistin or other antibiotics depends on the pharmacokinetics and pharmacodynamics of these molecules in vivo. Therefore, future studies should be performed to correlate the safety, efficacy, and pharmacokinetic parameters of these combinations.

## Figures and Tables

**Table 1:** Comparative in vitro activity of antimicrobial agents against 60 isolates of *A. baumannii*.

Antibiotics	mg/L						Percent inhibited at CLSI breakpoints^a^		
	MIC range	MIC_50_	MIC_90_	MBC range	MBC_50_	MBC_90_	Susceptible	M.S.^b^	Resistant
CSA-13	1–16	2	8	1–32	2	16	—	—	—
Colistin	0.06–32	1	4	0.06–32	2	8	86	0	14
Tobramycin	0.3–160	1.25	80	0.3–160	2.5	160	45	0	55
Ciprofloxacin meropenem	0.3–80 16–128	80 32	160 64	0.6–160 16–256	80 64	160 128	5 0	0 0	95 100

^a^CLSI breakpoints for susceptible and resistant to colistin ≤2 mg/L and ≥4 mg/L; tobramycin ≤4 mg/L and ≥16 mg/L; ciprofloxacin ≤1 mg/L and ≥4 mg/L and meropenem ≤4 mg/L and ≥16 mg/L, respectively.

^b^M.S.: moderately susceptible.

**Table 2:** In vitro activity of CSA-13 and colistin combined with studied antibiotics against *A. baumannii* strains.

Antibiotic combinations	*n*	Number (%) of synergistic effects
CSA-13 + colistin	20	11 (55)
CSA-13 + tobramycin	20	7 (35)
CSA-13 + ciprofloxacin	20	8 (40)
colistin + tobramycin	20	9 (45)
colistin + ciprofloxacin	20	9 (45)
